# Bio-Functional Nanomaterials for Enhanced Lung Cancer Therapy: The Synergistic Roles of Vitamins D and K

**DOI:** 10.3390/jfb16090352

**Published:** 2025-09-19

**Authors:** Andreea Crintea, Camelia Munteanu, Tamás Ilyés, Ciprian N. Silaghi, Alexandra M. Crăciun

**Affiliations:** 1 Department of Molecular Sciences, University of Medicine and Pharmacy “Iuliu Hațieganu”, 400349 Cluj-Napoca, Romania; crintea.andreea@umfcluj.ro (A.C.); tamas.ilyes@umfcluj.ro (T.I.); acraciun@umfcluj.ro (A.M.C.); 2 Biology Section, University of Agricultural Sciences and Veterinary Medicine Cluj-Napoca, 400372 Cluj-Napoca, Romania; camelia.munteanu@usamvcluj.ro

**Keywords:** lung cancer therapy, vitamin D, vitamin K, bio-functional nanomaterials

## Abstract

Lung cancer remains a leading cause of cancer-related mortality worldwide, requiring the development of innovative and effective therapeutic strategies. Bio-functional nanomaterials, due to their unique physicochemical properties, offer a versatile platform for targeted drug delivery, controlled release, and multimodal therapies, thereby enhancing efficacy and reducing the systemic toxicity of conventional treatments. Independently, both vitamin D and vitamin K have demonstrated significant anti-cancer properties, including inhibition of proliferation, induction of apoptosis, modulation of angiogenesis, and attenuation of metastatic potential in various cancer cell lines and in vivo models. However, their clinical application is often limited by poor bioavailability, rapid metabolism, and potential for off-target effects. Specifically, by enhancing the solubility, stability, and targeted accumulation of fat-soluble vitamins D and K within tumoral tissues for improved lung cancer therapy, this review emphasizes the novel and cooperative role of bio-functional nanomaterials in overcoming these limitations. Future studies should focus on the logical development of sophisticated nanomaterial carriers for optimal co-delivery plans and thorough in vivo validation, aiming to convert these encouraging preclinical results into successful clinical treatments for patients with lung cancer.

## 1. Introduction

Lung cancer remains the main cause of cancer-related mortality worldwide, being an important challenge to global public health. A particular kind of cancer called lung cancer develops in the lungs when aberrant cells proliferate and grow out of control. These cells can harm nearby healthy tissue and develop into cancers. Smoking is the most significant risk factor for cancer, which is the leading cause of cancer deaths globally. However, additional risk factors such as exposure to radon gas, air pollution, and secondhand smoke can also affect non-smokers [1]. Research on the effects of vitamins D and K on lung cancer is still ongoing. Although there has been some evidence that vitamin D has a preventive impact and that higher levels of the vitamin are linked to a lower risk of lung cancer, these results remain inconclusive [2].

Large-scale randomized controlled trials, which are regarded as the gold standard in medical research, have produced a variety of results. Several of these studies have not demonstrated that vitamin D supplements significantly reduce the risk of lung cancer [3]. This disparity could be caused by several factors, such as the vitamin D dosage, the length of time the subjects were taking supplements, their individual genetic composition, and variations in their initial vitamin D levels. A person who has a severe vitamin D shortage, for example, may react differently to supplements than someone who has appropriate amounts. Although vitamin D plays a critical role in bone health, its potential to prevent or treat lung cancer remains a matter of debate [4].

The relationship between vitamin K and lung cancer is still being studied. The majority of the data supporting vitamin K’s function comes from preclinical research and a small number of observational studies, in contrast to vitamin D, which has been thoroughly examined [5]. The capacity of vitamin K to cause cancer cells to undergo apoptosis, or programmed cell death, is one of its hypothesized anti-cancer mechanisms. Additionally, it might disrupt cell signaling pathways that are essential for the growth and survival of tumors [5].

Certain types of vitamin K, namely vitamin K2, have been demonstrated in some lab studies to dose-dependently prevent the growth of lung cancer cells. Though encouraging, these findings have not yet been confirmed in extensive human testing [6].

In this regard, by ensuring that vitamins D and K reach their target in the appropriate concentration at the right time, nanomedicine could transform the potential but currently unreliable benefits of these nutrients into a more accurate and successful treatment. However, further study and clinical trials are required to fully realize this potential, as this is still a developing sector.

The conflicting results of in-depth studies on the anti-cancer effects of vitamins D and K, especially with regard to lung cancer, point to a significant barrier to their therapeutic use: their intrinsic restrictions on bioavailability and targeted administration. In this sense, nanomedicine offers a compelling solution. By utilizing creative delivery techniques, nanotechnology can circumvent these limitations and help these vitamins realize their full therapeutic potential in a more regulated and effective manner.

Although significant advancements in diagnostic tools and therapeutic strategies have occurred over the past few decades, the overall survival rate for lung cancer patients remains low. This is due to various factors, including late-stage diagnosis, aggressive metastatic potential, and the development of drug resistance [7]. Conventional treatment methods, such as surgical resection, chemotherapy, radiation therapy, and more recently, targeted molecular therapies and immunotherapies, have enhanced patient outcomes. However, these approaches often come with several drawbacks, including systemic toxicity, severe side effects, and inadequate therapeutic effectiveness. These issues are particularly due to non-specific drug distribution and poor accumulation of treatment agents at tumor sites [8,9]. These pressing challenges underline the need for innovative and effective treatment strategies that can precisely target cancer cells while minimizing harm to healthy tissues [10,11].

Latest advances in the biomedical science field of nanotechnology have created new avenues for the treatment of cancer, especially in the area of nanomedicine. Engineered at the nanoscale (usually 1–100 nm), bio-functional nanomaterials offer a remarkable platform for revolutionizing therapeutic interventions and medication delivery [12]. Their properties, including high surface-to-volume ratios, adaptable size and shape, and the ability to easily adapt surface properties, enable precise targeting, controlled drug release, and the incorporation of multiple therapeutic or diagnostic features into a single system [13,14]. These features offer great potential to boost how well drugs work, improve how they are processed in the body, and—most importantly—reduce the widespread side effects often seen with traditional chemotherapy [15].

According to preclinical studies, specific fat-soluble vitamins, such as vitamin D and vitamin K, may have anti-cancer effects. Apart from their physiological functions in bone metabolism and coagulation, these vitamins have demonstrated notable capacities in preventing the growth of cancer cells, triggering apoptosis, regulating angiogenesis, and reducing the likelihood of metastasis in various cancer cell lines and in vivo models [6,16]. However, their direct clinical application as therapeutic agents is severely constrained by inherent limitations such as poor aqueous solubility, rapid metabolism, low bioavailability, and the potential for off-target toxicities (e.g., hypercalcemia with vitamin D) [17].

This review addresses a significant gap in the literature by concentrating on the synergistic potential of bio-functional nanomaterials in overcoming the inherent limitations of vitamins D and K, especially in the context of lung cancer treatment. Although research on the relationship between vitamins and cancer and nanotechnology is still in its infancy, there is not yet a thorough examination that emphasizes the intricate relationships between these fat-soluble vitamins and state-of-the-art nanomaterial delivery systems for the treatment of lung cancer. Our goal is to demonstrate how nanomaterials offer a new and up-and-coming method to fully unlock the therapeutic potential of these vitamins by encapsulating and delivering them in a targeted and controlled manner. Analyzing the anti-cancer potential of vitamins D and K in the context of lung cancer, identifying their clinical limitations, and how these can be overcome by bio-functional nanomaterials (liposomes, polymeric nanoparticles, micelles, inorganic nanoparticles) could open the door to more effective and less damaging methods of therapy for patients with lung cancer.

This is the first comprehensive review combining the roles of vitamins D and K with bio-functional nanomaterials specifically for lung cancer therapy. We have also discussed the synergism between vitamins D and K, as well as their co-delivery using the same platform and their integration with modern cancer therapies.

## 2. Targeted Nanomaterials in Cancer Therapy: Principles and Applications

Nanomedicine enables the delivery of drugs with extreme precision and the integration of various therapeutic modalities, significantly altering the way cancer is treated. Nanoscale-engineered bio-functional nanomaterials are made especially to interact with biological systems in a controlled way, providing special qualities that get around the inherent drawbacks of conventional cancer treatments [18].

### 2.1. Fundamental Principles of Nanomedicine in Oncology

The effectiveness of nanomedicine in cancer therapy largely depends on its ability to deliver therapeutic agents specifically to tumor sites while minimizing exposure to surrounding healthy tissues. This selective accumulation is governed by several fundamental principles that exploit the distinct pathophysiological characteristics of the tumor microenvironment [19].

One primary principle is the enhanced permeability and retention (EPR) effect, often referred to as passive targeting. This phenomenon capitalizes on the abnormal and leaky vasculature commonly found in solid tumors. Unlike well-organized, tightly structured blood vessels in healthy tissues, tumor blood vessels are typically characterized by rapid, disorganized angiogenesis. This results in vessels with structural abnormalities, including larger intercellular gaps (ranging from 100 nm to 2 μm). Consequently, nanoparticles, by their optimal size (generally between 20 and 200 nm), can exit through these large gaps and passively accumulate within the tumor interstitial space. Furthermore, the lymphatic drainage system within tumors is often dysfunctional or absent, which means that once nanoparticles have accumulated, they are retained for longer periods. As a result, the drug concentrations at the tumor site are noticeably higher and last longer than those in the systemic circulation [20,21]. This passive accumulation is crucial for improving the therapeutic index and reducing the systemic toxicity typically associated with conventional chemotherapeutics [22].

To improve the selectivity and cellular uptake of nanomaterials, active targeting techniques are used in addition to passive accumulation. This is performed by adding specific ligands to the nanocarriers’ surface that have a strong affinity for receptors that are overexpressed on the surface of tumor-associated stromal cells or cancer cells [23]. These ligands can be diverse, ranging from antibodies [e.g., those targeting human epidermal growth factor receptor 2 (HER2) or epidermal growth factor receptor (EGFR), common in lung cancer] to peptides [e.g., short amino acid sequences, Arginine-Glycine-Aspartic acid, acting as a cell adhesion motif (RGD peptides)] that bind to integrins, aptamers, or even small molecules (e.g., folate, transferrin). Upon recognizing and binding to their specific target receptors, the ligand-receptor interaction often triggers receptor-mediated endocytosis, leading to the efficient internalization of the nanocarrier and its therapeutic payload directly into the target cancer cell [24,25]. Active targeting aims to enhance cellular uptake beyond what the EPR effect alone can achieve, address issues of heterogeneous EPR in certain tumors, and pave the way for more personalized cancer therapies based on a patient’s tumor-specific biomarker expression profiles [26].

One of nanomedicine’s primary benefits is its ability to release drugs in a controlled, stimulus-responsive manner. Drug delivery may be precisely targeted and timed thanks to this cutting-edge feature. To help maintain stable drug levels in the body and potentially reduce the frequency of doses required, the release can be precisely regulated to provide a slow, continuous flow of medication [27,28]. More advanced nanomedicine designs use stimuli-responsive systems, where the drug is released only when triggered by specific conditions in the tumor environment. These internal (or endogenous) triggers can include the slightly acidic pH of tumor tissue, high levels of certain enzymes like matrix metalloproteinases or proteases, low oxygen levels (hypoxia) in poorly vascularized areas, or changes in redox balance—such as elevated glutathione levels inside cancer cells. External (or exogenous) triggers, like light (used in photothermal or photodynamic therapy), magnetic fields, or ultrasound, can also be used to release the drug with high precision. This level of control helps limit drug exposure to the rest of the body, reduces drug breakdown before it reaches the tumor, and ensures that the treatment is delivered exactly where it is needed most [29,30].

Furthermore, theragnostic is embodied by bio-functional nanomaterials, which are especially suited for fusing diagnosis and treatment. These sophisticated nanoparticles can be designed to deliver multiple therapeutic agents simultaneously, enabling more effective combination therapies, such as the co-administration of genetic therapy agents and cancer chemotherapy drugs [14,31]. Beyond therapy, they can integrate diagnostic imaging agents [e.g., magnetic resonance imaging (MRI) contrast agents, fluorescent dyes, radioisotopes]. This integration enables real-time visualization of tumor accumulation, dynamic tracking of drug release, and continuous monitoring of therapeutic response, all within a single system [30]. The theranostic approach offers a pathway towards personalized medicine, providing clinicians with vital real-time information to guide treatment decisions, assess efficacy dynamically, and optimize patient outcomes [32].

Aside from that, nanomedicine is opening up new areas like gene therapy. Nanoparticles can directly transfer genetic material into cancer cells, blocking genes that promote tumor growth or treatment resistance, as opposed to only delivering drugs. For example, a small piece of RNA that is meant to silence a specific gene can be turned into a nanoparticle [33]. This novel approach makes cancer cells more susceptible to treatment in a highly selective and efficient manner when paired with active targeting (such as RGD peptides that attach to integrins on tumor cells). These illustrations show how nanomedicine is developing from a straightforward delivery method into an advanced, multifunctional technology that can detect, treat, and track cancer with previously unheard-of accuracy [34].

We summarized the general principles and applications of bio-functional nanomaterials in oncology in Table 1.

### 2.2. Types of Targeted Nanomaterials for Lung Cancer Drug Delivery: Advantages and Disadvantages

The unchecked proliferation of cells in lung tissues is a hallmark of lung cancer. It can be generally divided into two types: small cell lung cancer (SCLC) and non-small cell lung cancer (NSCLC), which make up the majority of cases [46]. Lung cancer is difficult to treat with traditional medicines because of its physiological features, which include a highly vascularized but disordered tumor microenvironment [47]. Lung cancer cells have abundant HER2 or EGFR on their surface, but healthy cells do not. They are, therefore, perfect candidates for medication delivery using nanoparticles. In order to deliver therapeutic medications directly to a tumor while limiting damage to healthy tissue, nanoparticles must be engineered for active targeting. This can be accomplished by altering nanoparticles so that their surface enables them to bind to particular receptors, like EGFR or HER2, that are overexpressed on cancer cells. By ensuring that the therapy is administered where it is most needed, this precise targeting mechanism increases therapeutic efficacy and minimizes side effects.

Nanoparticles must be designed with particular characteristics to get beyond these physiological barriers in order to be used as an effective treatment. Their main goal is to minimize damage to healthy tissues while delivering therapeutic medicines straight to the tumor. Because of the enhanced permeability and retention (EPR) effect, nanoparticles must be small enough (usually between 20 and 200 nm) to pass through blood arteries and gather in the leaky tumor microenvironment [22].

Additionally, they must be made with a surface that can be altered for active targeting, which enables them to attach to receptors that are overexpressed on cancer cells, such as EGFR or HER2 [12]. The most popular way to provide systemic nanoparticles is by intravenous injection; however, intrapleural injection or inhalation can accomplish targeted distribution. For these delivery systems to be as effective as possible, they need to be able to release drugs in a regulated, stimulus-responsive manner and remain stable in the bloodstream [48].

A diverse array of nanomaterial platforms has been extensively explored for cancer therapy, each possessing distinct characteristics, advantages, and limitations for encapsulating and delivering drugs. The selection of an appropriate nanocarrier depends heavily on the properties of the therapeutic agent, the specific type of lung cancer, and the desired drug release profile. When designing bio-functional nanomaterials for lung cancer, considerations often include pulmonary delivery routes (e.g., intravenous vs. inhalation), overcoming the natural defense mechanisms of the lung (e.g., mucociliary clearance and alveolar macrophages), and targeting specific lung cancer biomarkers [12,49,50].

#### 2.2.1. Liposomes

Liposomes are highly versatile self-assembling spherical vesicles, typically ranging from 50 nm to several hundred nanometers in diameter. They are composed of one or more concentric lipid bilayers that encapsulate an aqueous core [51]. The main components often include phospholipids (such as phosphatidylcholine and phosphatidylethanolamine) and cholesterol, which play a crucial role in regulating membrane fluidity, permeability, and overall structural stability. Their unique structure allows for the encapsulation of both hydrophilic drugs within their aqueous core and lipophilic drugs (like vitamins D and K) within their lipid bilayer, making them exceptionally versatile [52,53].

Considering their lipid components are found naturally in the body, liposomes have exceptional biocompatibility and biodegradability, resulting in low immune reactions and long-term toxicity concerns. This is particularly important for repeated administrations and the delicate lung environment [54]. Their versatile drug encapsulation capacity, allowing for both hydrophilic and lipophilic payloads, effectively enhances the solubility of hydrophobic drugs and protects them from premature enzymatic degradation in vivo. Furthermore, liposomes offer tunable surface modifiability. PEGylation (the process of modifying molecules with polyethylene glycol) can create “stealth” liposomes that evade immune recognition and prolong circulation for intravenous delivery, increasing passive tumor accumulation [55]. PEGylation can trigger an immunological reaction even if it renders liposomes “stealthy” to increase stability and extend circulation.

This prolongs the liposome’s duration in the bloodstream by forming a hydrophilic barrier that stops the immune system from marking it for elimination [56]. Additionally, by keeping the liposome from clumping together, this barrier increases its stability and solubility. In addition to lowering toxicity, PEGylation facilitates more targeted medication delivery by decreasing non-specific interactions with healthy cells. PEGylation’s immunogenicity is a major drawback in spite of these benefits [57]. Repetitive use of PEGylated liposomes may cause the body to produce antibodies against PEG. As a result, the following dosages are rapidly removed from the bloodstream, decreasing the treatment’s effectiveness. This process is known as accelerated blood clearance, or ABC [58]. In addition to protecting the liposome, the same PEG coating also serves as a barrier, which may prevent cancer cells from absorbing it. In other cases, PEGylation may impair the structure of the liposome, which could lead to an early drug leak [59]. For active targeting, specific ligands (e.g., antibodies to EGFR, peptides, or folate) can be conjugated, enabling selective binding and internalization by lung cancer cells. This is highly relevant for inhaled delivery, where surface modifications can improve mucopenetration and cellular uptake. Importantly, liposomes have an established clinical precedent, with several approved formulations for cancer treatment, indicating a clearer and potentially faster pathway for regulatory approval of new liposome-based formulations in lung cancer [55,60,61,62].

Notwithstanding their benefits, liposomes have a number of drawbacks. They may be prone to early drug release and have poor in vivo stability. These systems may occasionally leak their drug cargo or be broken down by bloodstream enzymes, which could result in an early release of the medication. Less medication reaches the lung tumor as a result, which may reduce its effectiveness and raise the possibility of unintended side effects in other body areas [63]. Challenges in manufacturing consistency and scalability are also significant. Producing liposomes with consistent size distribution, lamellarity, and drug loading efficiency on a large scale remains a complex process, which impacts reproducibility and cost-effectiveness. For inhaled delivery, liposomes can be rapidly internalized and cleared by alveolar macrophages, significantly reducing their retention time in the lung and local therapeutic effect [64,65,66]. Lastly, in aggressive lung cancer types with dense stroma, the relatively large size of some liposomes might lead to limited tumor penetration, affecting drug distribution throughout the tumor mass [39].

#### 2.2.2. Polymeric Nanoparticles

Polymeric nanoparticles are solid colloidal particles, typically ranging from 10 to 1000 nm in size. They are formed by encapsulating therapeutic agents within a matrix of biodegradable and biocompatible polymers, which can be natural (e.g., chitosan, albumin) or synthetic (e.g., Poly Lactic-co-Glycolic Acid, PEG, Poly(ε-caprolactone)). The drugs can be uniformly dispersed within the polymer matrix, adsorbed onto the surface, or chemically conjugated to the polymer chains, providing substantial flexibility in design [67,68].

In terms of design and release kinetics, polymeric nanoparticles provide remarkable versatility. Precise control over size, shape, surface chemistry, and drug encapsulation is made possible by the selection and manufacturing of polymers, resulting in customized release profiles that range from rapid burst to weeks of continuous release. Because it prevents them from breaking down too quickly, this prolonged release is beneficial for preserving therapeutic concentrations of substances with short half-lives, including vitamins D and K [69,70]. These materials are highly biocompatible and break down in a controlled way into non-toxic byproducts, which helps prevent long-term buildup and reduces the risk of toxicity in lung tissue—an important safety factor for ongoing cancer treatment. For inhaled delivery, surface modifications like PEGylation can enhance their ability to penetrate the lung’s mucus layer and stay stable, leading to better local drug delivery [71,72]. Another key advantage is their high drug-loading capacity and ability to carry multiple agents at once—such as vitamins D and K, along with chemotherapy or gene therapy drugs. This enables combination therapies that work together to target multiple cancer pathways, which may help overcome drug resistance and improve treatment outcomes [73].

The potential for initial burst release, in which a portion of the encapsulated medication is released too quickly, can occur with polymeric nanoparticles despite their adaptability. This could result in systemic exposure and off-target consequences before the drug reaches the tumor. Reproducible synthesis at scale continues to pose challenges, as it can be difficult to achieve uniform drug distribution, shape, and particle size during large-scale manufacture, which impacts clinical translation [69,74]. While generally biocompatible, certain polymer compositions can still lead to the potential for immune recognition and clearance by macrophages, reducing their circulation time and tumor accumulation. Furthermore, some manufacturing processes involve organic solvents, requiring rigorous purification to ensure the complete removal of residual solvent toxicity [12,75].

#### 2.2.3. Inorganic Nanoparticles

This class of nanomaterials includes nanoparticles synthesized from metals (e.g., gold, silver), metal oxides (e.g., iron oxide, silica, titanium dioxide), or carbon-based structures (e.g., carbon dots, graphene oxide). These materials are prized for their unique physical, optical, electrical, and magnetic properties that extend their utility beyond simple drug delivery, often functioning as theranostic agents that combine diagnostic capabilities with therapeutic functions [76,77].

Inorganic nanoparticles offer diverse functionalities and theranostic capabilities. Gold nanoparticles, for example, possess unique surface plasmon resonance properties, enabling photothermal therapy for localized lung cancer ablation, photoacoustic imaging, and excellent computed tomography contrast agents for improved tumor visualization. Iron oxide nanoparticles are exceptional MRI contrast agents and can be used for magnetic hyperthermia or magnet-guided drug delivery. Mesoporous silica nanoparticles provide high drug-loading capacity and controlled release [78,79]. They generally exhibit high stability and robustness, protecting encapsulated drugs from degradation in vivo. Their properties, including size, shape, and optical and magnetic characteristics, are controllable during synthesis, allowing for highly customized designs optimized for specific lung cancer applications, such as enhanced tumor penetration or specific imaging modalities. This precise control also translates to a potential for high drug loading, especially for mesoporous structures, which is advantageous for delivering potent, low-dose therapeutics [13,80,81,82].

A significant concern for inorganic nanoparticles is long-term toxicity and biodegradability. Despite promising biocompatibility, questions remain about the ultimate fate of non-biodegradable or slowly biodegradable types in vivo. Chronic accumulation in organs such as the liver, spleen, and, specifically, the lungs can lead to inflammatory responses or granuloma formation, posing a significant hurdle for clinical translation. There is also a potential for an immune response and clearance if not properly surface-modified, which reduces their efficacy [82]. Challenges in scalable, reproducible synthesis persist, making large-scale, Good Manufacturing Practices (GMP)-compliant production technically complex and expensive. Lastly, while different particles can be made stimuli-responsive, many have limited drug-release trigger specificity compared to advanced polymeric systems, which can precisely release drugs based on subtle environmental changes [12].

#### 2.2.4. Micelles

Micelles are dynamic, core–shell nanoparticles, typically ranging from 10 to 100 nm in diameter. They spontaneously form through the self-assembly of amphiphilic block copolymers (molecules with both hydrophilic and hydrophobic segments) when their concentration surpasses the critical micelle concentration (CMC) in an aqueous solution. The hydrophobic segments aggregate to form a core that serves as a reservoir for encapsulating water-insoluble (lipophilic) drugs, while the hydrophilic segments extend outwards, forming a steric stabilization layer (corona) that reduces non-specific interactions with biological components, often imparting “stealth” properties [83,84].

Micelles offer exceptional solubilization of hydrophobic drugs, such as vitamins D and K, which dramatically improve their aqueous solubility and systemic bioavailability. This is crucial for achieving rapid and predictable drug concentrations in lung cancer patients via intravenous administration. The hydrophilic shell, typically PEG, contributes to prolonged systemic circulation and an enhanced EPR effect by reducing non-specific interactions with blood components and preventing rapid immune clearance. This extended circulation time increases their passive accumulation in lung tumors. Their small size, which enhances tumor penetration (typically <100 nm), is advantageous, allowing them to penetrate deeper into the interstitial space of lung tumors and potentially overcome diffusion barriers [83,85]. Additionally, micelles are generally easy to prepare and scale up through simple self-assembly methods, which can contribute to lower production costs compared to more complex nanostructures [83].

Micelles’ low in vivo stability and early dissociation are the primary concerns. As dynamic, non-covalently formed structures, they are likely to prematurely dissociate when diluted in the bloodstream if concentrations fall below their critical molecular concentration. This makes it difficult to maintain steady medication levels in the lung because it causes the encapsulated drug to release quickly and non-specifically throughout the body, thus decreasing tumor targeting effectiveness and raising systemic toxicity [86,87]. This also results in drug loading capacity and retention challenges, as the dynamic equilibrium can lead to early drug leakage, especially at sub-CMCs. Consequently, the potential for rapid clearance of the free drug is high when micelles dissociate, as the released hydrophobic drug might be quickly cleared or bind non-specifically to plasma proteins, limiting its therapeutic efficacy at the tumor site [83,88]. Finally, their efficacy and stability are highly dependent on CMC, requiring careful design of block copolymers with very low CMCs to ensure stability in the highly diluted environment of the bloodstream [87].

## 3. Vitamin D: Anti-Cancer Mechanisms and Clinical Challenges

Vitamin D is an essential fat-soluble vitamin that comes in two primary forms: vitamin D2 from plants and vitamin D3 from sunshine. The regulation of calcium and phosphate depends on it [89]. The liver is where the body first converts vitamin D into calcidiol, which is the primary form that is in circulation. Following that, it is transformed into calcitriol, its active hormonal form, in the kidneys. In clinical settings, bone-related conditions are treated with synthetic calcitriol [90]. Beyond its role in calcium homeostasis and bone health, vitamin D, particularly its active form 1,25(OH)_2_D3 (calcitriol), has emerged as a molecule with significant anti-cancer properties [91].

### 3.1. Anti-Cancer Mechanisms of Vitamin D

The anti-cancer actions of vitamin D are primarily mediated through its binding to the vitamin D receptor (VDR), a nuclear receptor widely expressed in various tissues, including lung epithelial cells and lung cancer cells. Upon binding, the 1,25(OH)_2_D3-VDR complex translocates to the nucleus, where it acts as a transcription factor, modulating the expression of numerous genes involved in critical cellular processes [92,93].

Calcitriol has been found to slow down the growth of different cancer cells, including lung cancer, mainly by stopping their cell cycle at the G0/G1 phase. This is achieved by upregulating cyclin-dependent kinase (CDK) inhibitors, such as p21WAF1/CIP1 and p27Kip1, which prevent the progression of the cell cycle and thereby hinder the uncontrolled cell division characteristic of malignancy [94].

Furthermore, vitamin D actively promotes programmed cell death (apoptosis) in cancer cells, a crucial mechanism for tumor regression. It can upregulate pro-apoptotic proteins (e.g., Bax, Bak) and downregulate anti-apoptotic proteins (e.g., Bcl-2, Bcl-XL), thereby shifting the cellular balance towards apoptosis. Moreover, it can induce the activation of caspase pathways, which are central to the execution of apoptosis [95,96].

In addition, vitamin D’s anti-angiogenic properties are crucial because angiogenesis —the development of new blood vessels that carry nutrients and oxygen—is a significant factor in tumor growth and metastasis. It accomplishes this by suppressing endothelial cell migration and proliferation, as well as downregulating pro-angiogenic factors such as vascular endothelial growth factor (VEGF) and its receptors [97,98].

Calcitriol also actively interferes with various stages of the metastatic process. It reduces the invasive and migratory capacity of cancer cells by modulating adhesion molecules such as E-cadherin and inhibiting matrix metalloproteinases (enzymes crucial for tissue breakdown and tumor dissemination) [99].

Furthermore, in certain cancer cell types, vitamin D can induce terminal differentiation, prompting malignant cells to adopt a more mature, less aggressive, and often non-proliferative phenotype. This vitamin also demonstrates the ability to modulate immune responses within the tumor microenvironment, potentially enhancing anti-tumor immunity by influencing the activity of various immune cells [100].

In vitro studies using various lung cancer cell lines (e.g., A549, NCI-H460, H1299) have demonstrated the anti-proliferative and pro-apoptotic effects of calcitriol. In vivo xenograft models in immunocompromised mice have confirmed these findings, showing reduced tumor growth and metastatic burden upon systemic administration of vitamin D analogs [101].

In order to maximize the therapeutic benefits of calcitriol while reducing its risk of hypercalcemia (high blood calcium), vitamin D analogs were employed in the study. Because they can more effectively limit tumor growth, induce cell death, and block the production of new blood vessels, these synthetic chemicals are structurally changed to be more effective at targeting cancer cells. Furthermore, some analogs have better pharmacokinetics, which means that their blood half-life is greater, allowing for a longer-lasting anti-cancer impact. In the end, employing these analogs allows for the clinical testing of high therapeutic dosages of vitamin D-based treatments without the possibility of serious harm [102].

### 3.2. The Vitamin D Puzzle in Lung Cancer Patients

Vitamin D metabolism disorders are common in people with lung cancer [103]. A strong link between smoking and lung cancer has been firmly established by several studies [104]. Smokers’ levels of circulating 25-hydroxyvitamin D (calcidiol), the primary form of the vitamin that is stored, are consistently lower than those of non-smokers. The leading cause of this is smoking’s capacity to raise 24-hydroxylase activity, an enzyme that degrades active vitamin D. Consequently, less calcitriol, the vitamin’s physiologically active form, is available to the body [105]. Because smokers’ calcitriol’s well-established anti-cancer effects—such as inducing cell death and reducing cell growth—are weakened, this is a serious problem. In addition to directly harming lung cells, the chemicals in cigarette smoke also disrupt vitamin D metabolism, which erodes the body’s defenses. This link results in a lethal synergy. Because the vitamin’s anti-cancer capabilities are not properly exploited, the consequent vitamin D deficiency may worsen a person’s prognosis [106]. Thus, smoking increases a person’s risk of developing lung cancer by introducing carcinogens into the lungs and undermining a vital defense system. Smoking is a significant risk factor for the condition, as this dual-action impact demonstrates [107]. The molecular mechanism has shown that 24-hydroxylase is expressed in response to benzo[a]pyrene (BaP), a primary toxic substance produced during cigarette combustion [108]. However, 24-hydroxylase’s catalytic activity rises with increased expression, which speeds up the catabolic process of 1,25(OH)_2_D3 and eventually produces inactive 24,25-(OH)_2_D3. CYP24A1 is often overexpressed in tumor tissues, as Shiratsuchi et al. showed. Circulating bioactive vitamin D concentrations decrease as a result of this increased expression, which makes it easier for bioactive 1,25(OH)_2_D3 to be converted into its inactive metabolite [109]. Furthermore, studies on patients with lung carcinoma revealed that serum 25-(OH) D levels gradually decrease in tandem with the progression of the tumor stage in patients with pulmonary malignancy [103]. This metabolic anomaly highlights the potential practical uses of vitamin D research in patients with lung cancer by suggesting that they may benefit more from vitamin D-containing treatment approaches.

### 3.3. The Role of Vitamin D in Reprogramming the Immunosuppressive Tumor Microenvironment for Enhanced Immunotherapy

T cells are a key element and are essential for tumor immunity. Because they can recognize and destroy cancer cells that directly express tumor antigens, cytotoxic T lymphocytes (CTLs) are particularly important. Immune checkpoint Inhibitors (ICIs) are a family of medicinal drugs that work by blocking immunological checkpoint molecules to increase the immune response. The condition of immune cells in the tumor microenvironment (TME) has a significant impact on the efficacy of ICIs. By encouraging T-cell development and activation and regulating Treg cytokine production, vitamin D enhances immunosuppression in TME. ICIs are more effective as a result of this activity. As a result, vitamin D and ICIs might work in concert to improve lung cancer patients’ prognosis and treatment results [110]. Essentially, vitamin D helps optimize the immune system for greater efficacy rather than merely enhancing immunosuppression. Vitamin D may work in concert with ICIs to enhance the prognosis and treatment results for patients with lung cancer by influencing T-cell activity and directly downregulating checkpoint molecules.

In the TME, continuous antigenic stimulation causes CD8+ T cells to progress toward an exhausted state progressively. The elevated expression of co-inhibitory receptors, such as PD-1, TIGIT, and TIM-3, demonstrates this mechanism. Because of their overexpression, which inhibits cellular activity, these receptors gradually lose their cytotoxic and proliferative qualities. These cells become tired CD8+ T effector (Tex) cells [111]. The co-inhibitory signaling cascade can be partially inhibited by immune checkpoint blockers (ICBs), which can save some PD-1-expressing cells from being fatigued or non-responsive. According to research findings, only recently exhausted T cells with relatively low PD-1 expression exhibit the potential for functional restoration in the setting of anti-PD-L1/PD-1 therapy. On the other hand, effector T cells that are over-exhausted exhibit reduced capacity to restore activity and greater levels of PD-1 expression in addition to other activation indicators (such as TIM-3 and TIGIT) [112]. Plasma vitamin D levels in lung cancer patients show a positive association with the expression of the co-stimulatory molecule CD28 and an inverse correlation with the expression of co-inhibitory immunological checkpoints. Gene regulation and epigenetic changes are the main mechanisms underlying this connection, which ultimately leads to an increase in CD28 expression and a decrease in PD-1 expression [113]. By blocking PD-1 expression, vitamin D can effectively restore T cell activity, which in turn improves the effectiveness of Immune Checkpoint Inhibitors (ICIs) in anti-tumor responses.

Vitamin D and the VDR can work together to increase Tregs, increase IL-10, and decrease pro-inflammatory cytokines like IL-17 and interferon-γ (IFN-γ). Vitamin D can rewire the TME’s immunosuppressive properties by altering the balance of Treg/Th17 cell populations. This produces an environment that is less conducive to tumor cell proliferation and improves the efficacy of immunotherapy [114]. VDR expression levels are substantially higher in tumor cells than in adjacent normal tissues, according to studies on lung adenocarcinoma. Furthermore, increased VDR expression is positively correlated with immune components within TME [115]. Therefore, vitamin D and VDR interaction may be a good way to change the immune response network in TME. This phenomenon can be explained by the fact that activated Tregs exhibit increased expression of the chemokine receptors CCR4, CXCR3, and CCR8, which promotes the directed migration of antigen-presenting cells and cytotoxic lymphocytes into the TME. This supports tumor immunotherapy by increasing cytotoxic activity against tumor cells and immune surveillance [116].

However, the action of vitamin D is more accurately characterized as immunomodulatory than immunosuppressive. Thebalance and the particular situation are crucial. Even while vitamin D has some effects on Tregs, it is thought to modify the immune response in the tumor microenvironment, ultimately making it more effective at fighting cancer [117].

Moreover, recent research suggests that vitamin D’s anti-cancer benefits are more widespread and beyond its role in increasing Tregs. It promotes the development of effector T-cells, which are in charge of destroying malignant cells [111]. It can make cancer cells more vulnerable to therapies like immune checkpoint inhibitors by downregulating immune checkpoint molecules like PD-1. Its wide-ranging anti-inflammatory properties may reduce the tumor’s growth-promoting potential. Essentially, the reason why scientists are investigating the use of vitamin D is that its overall immunomodulatory influence on the tumor microenvironment appears to tip the scales in favor of a more robust anti-tumor immune response, which increases the efficacy of other treatments [118].

In summary, vitamin D precisely modulates the TME through multiple regulatory pathways, transforming an immunologically “cold” TME into a receptive environment, in contrast to traditional anti-tumor agents that directly induce cytotoxic effects on tumor cells. As a result, it is essential for lung cancer immunotherapy. Patients with lung cancer who have become resistant to immunotherapy after using it for a long time may have new hope thanks to this innovative mechanism of action. Patients can once again benefit from immunotherapy because it enables the restoration of immune sensitivity to tumor cells [119].

**Figure 1:**
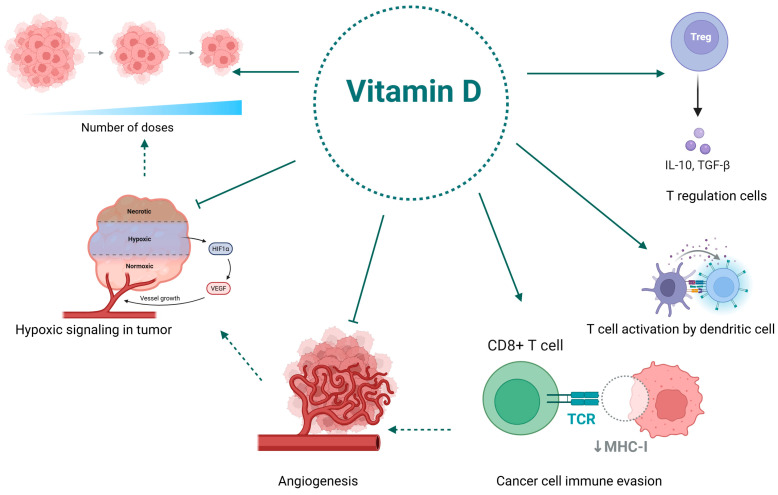
Synergistic effects of vitamin D and immune checkpoint inhibitors in lung cancer treatment. Vitamin D plays a role as an immunoregulator in lung cancer. By avoiding T-cell “exhaustion,” which is brought on by elevated concentrations of inhibitory checkpoints such as PD-1 in the tumor environment, it functions. Vitamin D helps T lymphocytes become more active by reducing PD-1 and increasing activating molecules, such as CD28. This changes the tumor environment from “cold,” unresponsive, to “hot,” active, increasing sensitivity to Immune Checkpoint Inhibitors (ICIs) and perhaps reviving therapy response in individuals who have become resistant.

The synergistic effects of vitamin D and immune checkpoint inhibitors in lung cancer treatment are depicted in Figure 1.

### 3.4. Clinical Limitations of Vitamin D

Despite compelling preclinical evidence, the direct clinical application of 1,25(OH)_2_D3 as a monotherapy for cancer is limited by several factors. As a lipophilic molecule, calcitriol exhibits poor solubility in aqueous biological fluids, leading to inefficient absorption from oral administration and challenges in formulating intravenous solutions. This results in low systemic bioavailability, meaning only a small fraction of the administered dose reaches the target tissues [120]. Furthermore, calcitriol is rapidly metabolized and cleared from the body by the enzyme 24−hydroxylase (CYP24A1) into inactive forms. This rapid degradation translates into a short half-life and therapeutic window, necessitating frequent and high dosing to maintain effective therapeutic concentrations [121]. The most significant dose-limiting toxicity of calcitriol is hypercalcemia. While therapeutic concentrations are beneficial for cancer cells, high systemic levels can lead to excessive calcium absorption from the gut and bone resorption, resulting in elevated blood calcium levels. Symptoms of hypercalcemia include nausea, vomiting, fatigue, kidney dysfunction, and, in severe cases, cardiac arrhythmias and coma [122]. This dose-limiting toxicity severely restricts the maximum tolerable dose of calcitriol that can be safely administered to cancer patients. Addressing these crucial limitations necessitates the development of advanced delivery strategies to fully harness vitamin D’s anti-cancer potential in a clinically viable manner [123]. The limitations of vitamin D are summarized in Table 2.

## 4. Vitamin K: Anti-Cancer Mechanisms and Clinical Challenges

Vitamin K, particularly its menaquinone (MK) forms, has recently garnered significant attention for its anti-cancer properties, distinct from its classical role in blood coagulation [124] and bone metabolism. Vitamin K is a fat-soluble vitamin for bone calcium control and blood coagulation. This product’s name is derived from the German word “Koagulationsvitamin.” Vitamin K1 (phylloquinone), which is found in plants, Vitamin K2 (menaquinone), which is created by gut bacteria, and Vitamin K3 (menadione), a synthetic version that is no longer used in clinical settings because of its toxicity, are the three main types. The body obtains K1 from food and K2 from fermented foods or gut microbes. It travels to the liver after being absorbed in the small intestine, together with lipids, since it is a fat-soluble [125].

Vitamin K is a cofactor for the gamma-glutamyl carboxylase (GGCX) enzyme. Specific proteins can bind calcium ions thanks to the modifications made by this enzyme. These VKDPs are necessary for two primary functions: bone health (e.g., osteocalcin) and blood coagulation (e.g., factors II, VII, IX, and X). In clinical circumstances, phytonadione, a synthetic form of K1, is utilized. It is frequently given to infants to stop vitamin K-deficiency bleeding (VKDB) and to adults to undo the effects of some anticoagulants, such as warfarin [126].

### 4.1. Anti-Cancer Mechanisms of Vitamin K

Vitamin K comprises two main forms: K1 (phylloquinone), found in green leafy vegetables, and MKs, primarily synthesized by gut bacteria and found in fermented foods and animal products. While both forms are crucial for physiological health, MKs have consistently demonstrated more pronounced anti-cancer effects in preclinical studies. The anti-cancer mechanisms attributed to vitamin K, particularly MKs, are multifaceted and include the induction of apoptosis [127]. Menaquinones have been shown to induce programmed cell death in various cancer cell lines, including those of lung origin, through mechanisms independent of their coagulation activity. This often involves the generation of reactive oxygen species (ROS), induction of oxidative stress, and the activation of specific caspase cascades (e.g., caspase-3, -8, -9), key enzymes in the apoptotic pathway [5].

Apart from triggering apoptosis, MKs also successfully stop cancer cells from growing by stopping essential signaling pathways and causing cell cycle arrest. For example, it has been shown that they interfere with the Wnt/β-catenin signaling pathway, which is frequently dysregulated in many malignancies and is known to promote cell survival and proliferation. Similarly to vitamin D, MKs possess strong anti-angiogenic properties that limit blood flow, a process necessary for tumor growth and survival. They achieve this by inhibiting the migration and proliferation of endothelial cells, as well as reducing the production of pro-angiogenic factors [128,129].

In different types of cancer, MKs have been shown to encourage malignant cells to mature into a less aggressive, slower-growing, and often more harmless form. This kind of “reprogramming” of cancer cells could be a powerful therapeutic strategy. Additionally, recent studies suggest that K vitamers can work alongside certain standard chemotherapy drugs, boosting their cancer-killing effects and potentially helping to overcome drug resistance [130].

In vitro studies have demonstrated the efficacy of MKs in inducing apoptosis and inhibiting proliferation in lung cancer cell lines. In vivo studies, although less extensive than for vitamin D, have also shown promising results in reducing tumor burden [129,131].

**Figure 2:**
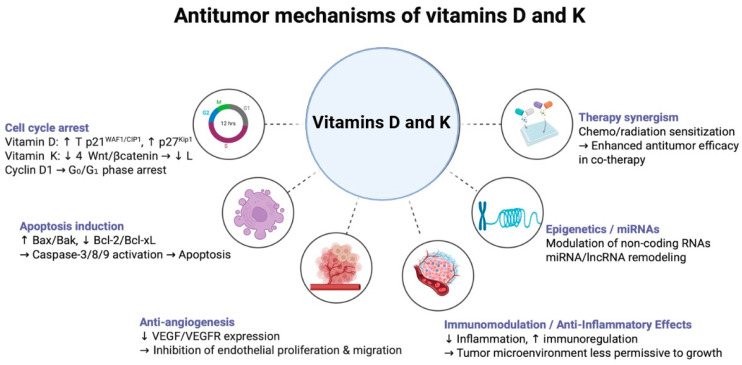
Antitumor mechanisms of vitamin D and K. Vitamin D exerts anti-cancer effects by reducing cell proliferation, promoting cell differentiation and apoptosis, and inhibiting angiogenesis, thereby limiting tumor growth and progression. Vitamin K exerts anti-cancer mechanisms by inducing apoptosis, programmed cell death in various cancer cell lines, arresting the cell cycle, and inhibiting key signaling pathways involved in cell growth.

The antitumor mechanisms of vitamin D and K are summarized in Figure 2.

### 4.2. The Relationship Between Vitamin K Status and Lung Cancer

The results regarding the association between vitamin K intake in thediet and the risk of cancer are preliminary and ambiguous. The relationships between vitamin K intake and the risk of various malignancies, such as prostate cancer, breast cancer, pancreatic cancer, and overall cancer, have been the subject of numerous studies [132,133]. Specifically, the association of dietary vitamin K intake and lung cancer risk could be explained by several potential mechanisms. Dietary vitamin K (phylloquinone) consumption has been documented to have an inhibitory effect on systemic inflammation by reducing the level of pro-inflammatory cytokines, particularly tumor necrosis factor-α and interleukin-6, a well-known factor for promoting the onset and development of cancer [134]. Moreover, vitamin K functions as a cofactor in the activation of protein C, a protein that depends on vitamin K and may restrict the extravasation of cancer cells [135,136].

The two forms of vitamin K2 (menaquinone) are menaquinone-4 (MK-4), which is a substance that is tissue-specifically generated from phylloquinone, and menaquinone-7 (MK-7), which has a longer half-life and may function similarly to MK-4 [137,138].

It has been demonstrated that vitamin K2 inhibits nuclear factor (NF)-κB activation by blocking the activities of protein kinase C (PKC)-α and ε kinases, which in turn prevents protein kinase D1 activation in cancer cells [139]. Although it is not found naturally, vitamin K3 (menadione) may be a byproduct of the intestinal breakdown of phylloquinone, which disrupts the mitochondrial membrane and produces reactive oxygen species, both of which have potent anti-cancer effects [140,141].

In conclusion, the primary naturally occurring vitamin K-driven components that inhibit the development of cancer are vitamins K2 and K3, which can be produced from vitamin K1 (phylloquinone). About 75–90% of vitamin K1 and 25% of vitamin K2 are found in the total vitamin K in the diet. Vitamin K3 (menadione) is produced by catabolizing a portion (5–20%) of the ingested vitamin K [142,143].

Vitamin K’s possible link to lung cancer has been the subject of numerous investigations. An extensive prospective cohort study found a negative correlation between dietary vitamin K intake and the incidence of lung cancer. This implies that the incidence of lung cancer was lower in those who consumed more vitamin K. There was a higher correlation between men and those who had smoked in the past. Although they did not establish causation, these observational studies did demonstrate a link. The results have drawn attention to the need for further study to understand the biochemical circuits at play fully [144].

More focused research has been conducted to gain a deeper understanding of the potential effects of vitamin K on lung cancer cells. Vitamin K has been shown in lab studies to have antitumoral effects. Vitamin K, particularly the MK-4 form of vitamin K2, has been shown to induce apoptosis, or programmed cell death, in various types of cancer cells, including those of the lung. Additionally, vitamin K has been shown to inhibit the growth of cancer cells by arresting the cell cycle at the G1 phase, thereby preventing cell division [144]. Other possible processes that have been investigated include the interruption of electron transport in the mitochondria of cancer cells, the production of reactive oxygen species (ROS), and the suppression of specific signaling pathways that promote cancer formation [145].

**Figure 3:**
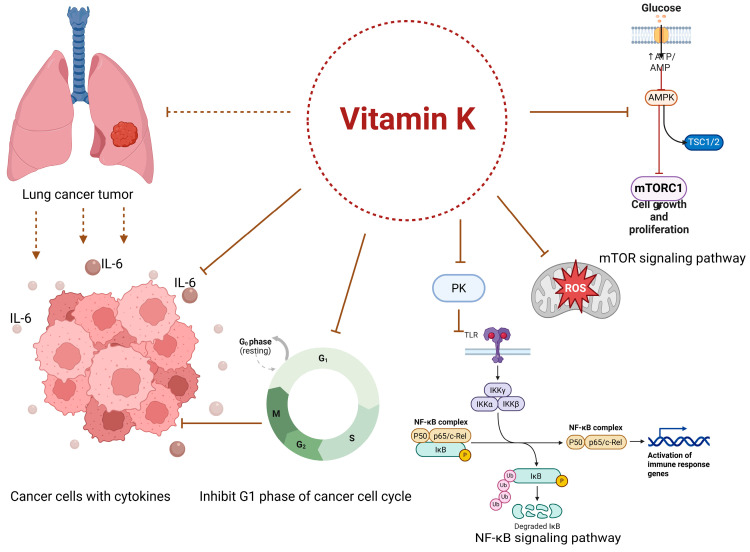
The relationship between vitamin K status and lung cancer. One proposed mechanism for its potential benefit is its ability to reduce systemic inflammation by lowering pro-inflammatory cytokines, such as IL-6. Vitamin K also acts as a cofactor for protein C, which may limit the spread of cancer cells. Different forms of vitamin K are believed to have potent anti-cancer effects. These include inducing programmed cell death (apoptosis), inhibiting reactive oxygen species (ROS), and blocking key signaling pathways, such as NF-κB, which promotes cell growth.

The relationship between vitamin K status and lung cancer is depicted in Figure 3.

### 4.3. The Tumor Microenvironment, Inflammation, and the Role of Vitamin K in Cancer Progression

The tumor microenvironment (TME) may undergo regular age-related alterations that could accelerate the spread of cancer metastases [146]. Cellular senescence may cause cancer or stimulate recurrence, hasten cancer metastasis, and lower the prognosis of cancer patients [147]. Cancer and the senescence process are linked to oxidative stress and inflammation. By triggering inflammatory pathways, oxidative stress can change healthy cells into malignant cells. Thus, knowing how vitamin K, cellular senescence, and cancer metastasis interact helps improve the prevention and treatment of cancer metastases and raises the survival rate of cancer patients.

The REDOX state may also impact the regulation of NF-κB. Furthermore, cell growth is stopped when NF-κB activation is inhibited. Tumor invasion can be facilitated by NF-κB, which can stimulate angiogenesis, tumor stromal cell proliferation, and malignant cell proliferation [147].

Numerous processes, such as enhancing angiogenesis, stimulating cell proliferation, and changing epigenetic state, are some of the ways that chronic inflammation contributes to cancer [148]. Several inflammatory chemicals are also intimately linked to the development of cancer [149]. On the other hand, persistent, chronic inflammation can change the tissue microenvironment, which can lead to the growth of cancer cells. Pro-inflammatory mediators may encourage angiogenesis. Additionally, the newly formed blood vessels may give tumor cells oxygen and nutrients, which would encourage the growth and metastasis of the tumor. However, by encouraging tumor invasion and metastasis, newly formed blood vessels can alter the extracellular matrix [150].

Pro-inflammatory cytokines, like interleukin-1 (IL-1) and tumor necrosis factor-α (TNF-α), can activate NF-κB in malignant cells, which activates anti-apoptotic genes and speeds up the disease’s progression. Recent studies have shown that MK can block the production of pro-inflammatory cytokines by interacting with the NF-κB signal transduction system. For example, MK induces anti-inflammatory characteristics in prostate cancer cells. Furthermore, MK can downregulate inflammatory genes, reduce cancer cell proliferation, and diminish angiogenic potential [151]. White blood cells and mast cells are transported to the injured area by the inflammatory process in the meantime. A “respiratory burst” caused by increased oxygen uptake increases ROS release and accumulation at the injured location [152]. It has been concluded that vitamin K helps the immune system in a variety of diseases, especially inflammatory and cancerous conditions. Vitamin K reduces inflammation by blocking the release of cytokines like IL-6 and the activity of nuclear factor kappa B (NF-κB) [153].

### 4.4. Clinical Limitations of Vitamin K

Similarly to vitamin D, the clinical utility of vitamin K as a direct therapeutic agent is blocked by several practical limitations. As a liposoluble vitamin, its poor bioavailability is a significant barrier. Optimal absorption often requires co-ingestion with dietary fats, and even then, significant inter-individual variability exists, making it challenging to achieve consistent and therapeutically relevant plasma concentrations [154,155]. Furthermore, rapid metabolism and liver clearance limit its systemic exposure. Vitamin K forms undergo relatively rapid hepatic metabolism and subsequent excretion. vitamin K has a short half-life, which means patients would need to take it often and at higher doses to keep the treatment effective. This frequent dosing can be inconvenient and is not always practical or ideal for long-term cancer care [156].

Ultimately, interference with anticoagulant medications represents the most critical clinical concern. Vitamin K is an essential cofactor for the γ-carboxylation of specific glutamic acid residues in vitamin K-dependent proteins, including several coagulation factors (II, VII, IX, X) [157]. Anticoagulant drugs, particularly vitamin K antagonists like warfarin, exert their effect by inhibiting vitamin K epoxide reductase, a crucial enzyme in the vitamin K cycle. High doses of exogenous vitamin K can directly counteract the action of warfarin, leading to a reduced anticoagulant effect and an increased risk of thrombotic events [158]. This well-established interaction requires careful monitoring of prothrombin time/international normalized ratio (PT/INR) in patients on these medications, severely limiting the concurrent therapeutic use of high-dose vitamin K [159]. Finally, developing stable and effective pharmaceutical formulations for systemic delivery, particularly via intravenous routes, is technically challenging due to the inherent lipophilic nature of vitamin K [160]. Without proper formulation, such as encapsulation in micelles, vitamin K tends to aggregate, precipitate, or be rapidly eliminated, which undermines its therapeutic effectiveness. These inherent physiological and pharmacological challenges underscore the urgent need for advanced delivery systems that can improve the bioavailability, stability, and targeted delivery of vitamin K to tumor sites, thereby enhancing its anti-cancer potential while minimizing systemic side effects and drug interactions [161,162]. Direct oral anticoagulants (DOACs), which are more modern and state-of-the-art anticoagulants, can be used to overcome this restriction. The vitamin K cycle is not disturbed by DOACs, in contrast to warfarin. Their approach, on the other hand, involves directly turning off specific blood coagulation proteins [163]. The two main categories of DOACs are direct thrombin inhibitors, such as dabigatran, and direct factor Xa inhibitors, including rivaroxaban, apixaban, and edoxaban. Vitamin K intake does not affect the anticoagulant action of these medications since they target distinct stages in the coagulation cascade. As a result, there is no drug-food interaction with vitamin K for patients on DOACs, and these drugs would not interfere with the therapeutic use of high-dose vitamin K [164]. This eliminates a key dietary and therapeutic limitation, which is a substantial advantage over warfarin.

The limitations of vitamin K are summarized in Table 2.

## 5. Nanomaterial-Enabled Delivery of Vitamins D and K

The inherent limitations of direct vitamin D and K administration underscore the critical need for advanced delivery strategies. Bio-functional nanomaterials offer an elegant and highly effective solution to overcome these challenges, enabling the synergistic utilization of these vitamins for enhanced lung cancer therapy.

### 5.1. Overcoming Bioavailability and Stability Issues

Nanomaterials provide a crucial approach to addressing the poor bioavailability and limited stability inherent to fat-soluble vitamins, such as D and K, which are essential for their effective therapeutic application in lung cancer. For fat-soluble vitamins, encapsulation within various nanomaterials, such as the hydrophobic core of polymeric nanoparticles, micelles, or within the lipid bilayer of liposomes, dramatically enhances their aqueous solubility [162]. This transformation into a solubilized nanoformulation enables systemic administration (e.g., intravenous injection), bypassing inefficient and variable oral absorption. Cross-linked nanomedicines also aim to address the issue of stability [165]. Consequently, a significantly higher and more consistent fraction of the administered vitamin dose reaches systemic circulation and the target tumor site [166,167,168].

Nanocarriers do more than just improve solubility—they serve as advanced protective barriers that encapsulate fragile vitamin molecules, shielding them from early degradation. Within the body, vitamins are vulnerable to enzymatic breakdown (such as by esterases or oxidases), oxidative damage, and rapid removal by the reticuloendothelial system, particularly in organs like the liver and spleen [169]. The physical barrier formed by the nanoparticle matrix—whether it is the polymer shell of a nanoparticle, the lipid bilayer of a liposome, or the core–shell structure of a micelle—greatly enhances the chemical and physical stability of these otherwise fragile compounds. This protection helps extend their half-life in the bloodstream, allowing for sustained therapeutic levels of the vitamins over a longer period [170,171]. This not only maximizes their therapeutic window at the tumor site but also potentially allows for reduced dosing frequency, thereby improving patient convenience and compliance, a key factor in long-term cancer therapy [27]. Deformable nanocarriers enhance these properties further by facilitating drug delivery to the targeted site [172].

However, it is a common misconception that all nanoparticles are readily absorbed by the reticuloendothelial system (RES), even though the RES is designed to remove foreign particles. Actually, sophisticated nanoformulation techniques are specifically designed to reduce this absorption drastically. The physical and chemical characteristics of the nanoparticles, which can be precisely designed to evade macrophage detection and clearance, are crucial to the efficacy of this strategy [173]. Surface charge engineering, size and shape control, and surface modification are the primary methods employed to lower RES uptake. Coating nanoparticles with hydrophilic polymers, such as polyethylene glycol (PEGylation), is a crucial technique that shields the particle from the RES by forming a “stealth” layer [174]. A surface with a neutral charge and a size that falls within a certain range—usually between 10 and 100 nm—can also be designed to lessen protein adsorption and clearance. By using these techniques, nanoparticles that can avoid the RES can be created, enhancing dispersion to specific regions and extending circulation times [175].

### 5.2. Achieving Targeted Accumulation and Reduced Off-Target Effects

A significant advantage of combining vitamins D and K with nanomaterials is precise tumor targeting, maximizing effectiveness while greatly reducing off-target side effects. This is achieved through a combination of passive and active strategies, alongside controlled release mechanisms responsive to the tumor microenvironment [12,176,177].

On one hand, passive targeting plays a foundational role. By designing nanoparticles within the optimal size range (typically 20–200 nm), they can passively accumulate in the leaky tumor vasculature via the EPR effect. This phenomenon leverages the distinct physiological abnormalities of solid tumors. Unlike healthy tissues, tumors undergo rapid and disorganized angiogenesis, resulting in newly formed blood vessels that are often structurally compromised. These vessels exhibit larger intercellular gaps (ranging from 100 nm to 2 μm, significantly larger than the tight junctions in healthy capillaries) and a defective lymphatic drainage system [21,35]. Nanoparticles, by their size, can readily extravasate through these large gaps and accumulate within the tumor interstitial space. Once in the tumor, the impaired lymphatic system prevents the rapid clearance of the nano-vitamin formulations, leading to their prolonged retention and a significantly higher local concentration compared to healthy tissues [12,178,179]. This passive accumulation alone reduces systemic exposure to the vitamins, thereby alleviating dose-limiting toxicities such as hypercalcemia associated with vitamin D and mitigating the risk of coagulation interference from vitamin K. However, the EPR effect can be heterogeneous across different tumors or even within the same tumor, making active targeting a valuable complementary strategy [180].

On the other hand, active targeting further refines specificity and aims to overcome the limitations of the heterogeneous EPR effect. Nanomaterials can be surface-modified with specific targeting ligands that are designed to selectively bind to receptors overexpressed on the surface of lung cancer cells or tumor-associated stromal cells, but are either absent or expressed at much lower levels on healthy cells [12,181]. Upon binding, the ligand-receptor interaction often triggers receptor-mediated endocytosis, leading to the efficient internalization of the nanocarrier and its therapeutic payload directly into the target cancer cell. This “lock-and-key” mechanism ensures that the nano-vitamin complexes are preferentially delivered to and taken up by malignant cells, leading to extremely high intracellular concentrations [182]. Common targeting ligands relevant for lung cancer include antibodies [such as anti-EGFR antibodies, which target EGFR, a receptor frequently overexpressed in non-small cell lung cancer (NSCLC)], peptides (like RGD peptides, which bind to αvβ3 integrins often upregulated on angiogenic endothelial cells and certain lung cancer cells, promoting cellular adhesion and internalization), small molecules (for instance, folate, which targets the folate receptor α often overexpressed in various lung cancer subtypes, enabling receptor-mediated endocytosis), and aptamers (nucleic acid sequences that can bind with high affinity and specificity to molecular targets) [177,183,184]. This active targeting strategy significantly increases intracellular uptake, maximizing the therapeutic effect of vitamins D and K within the lung cancer cells while sparing healthy lung tissue and other organs from high drug exposure [12,177,179].

Lastly, a key component of enhancing the therapeutic index is the controlled release of drugs within the tumor microenvironment. To make vitamin distribution even more accurate, smart nanoparticles can be designed to release their payload in a regulated manner in response to particular stimuli. The physical and chemical characteristics of the tumor environment are distinct from those of healthy tissues [20,185]. These differences can be exploited as triggers for drug release. For example, tumor cells often exhibit a higher rate of glycolysis, leading to lactic acid production and a slightly acidic extracellular pH (typically pH 6.5–6.8) compared to the physiological pH of blood (pH 7.4). pH-responsive nanoparticles can be engineered to swell or degrade, releasing their cargo preferentially in this acidic environment [186]. Likewise, overexpressed enzymes in the tumor microenvironment, such as matrix metalloproteinases or specific proteases (such as cathepsins), contribute to tumor invasion and metastasis. These enzymes can break down particular links in enzyme-responsive systems, causing localized vitamin release. Redox imbalances (e.g., elevated intracellular glutathione levels in cancer cells) and hypoxia (low oxygen levels), which frequently occur in solid tumors, are additional triggers [187,188,189]. Some nanomaterials can even be designed for temperature-responsive release, where an external heat source (e.g., focused ultrasound, near-infrared light) can induce drug release, offering precise spatiotemporal control. This localized and controlled release maximizes the therapeutic impact of vitamins D and K precisely where they are needed, minimizing their systemic exposure and thereby further reducing potential side effects and improving the overall safety profile of the therapy [190,191].

### 5.3. Synergistic Therapeutic Potential of Co-Delivery

The co-delivery of vitamins D and K within a single nanocarrier presents a compelling strategy for synergistic therapeutic effects, potentially enhancing their individual anti-cancer actions and overcoming resistance mechanisms. This approach leverages the distinct yet complementary biological pathways modulated by each vitamin, providing a more robust and comprehensive approach to targeting lung cancer cells than monotherapy [192,193]. We found four reasons that support the synergistic delivery of the two vitamins:

1. The complementary mechanisms of action of vitamins D and K provide a powerful rationale for their co-delivery. As discussed in previous sections, vitamin D primarily exerts its anti-cancer effects by inhibiting cell proliferation, inducing cell cycle arrest (predominantly at the G0/G1 phase via upregulation of CDK inhibitors like p21WAF1/CIP1 and p27Kip1), and promoting malignant cell differentiation [194]. Vitamin K, especially the menaquinones (MKs), is known to trigger apoptosis by generating reactive oxygen species, causing oxidative stress, and activating specific caspase pathways like caspase-3, -8, and -9. Both vitamins also play a role in preventing the formation of new blood vessels, though they may do so through different molecular targets or signaling pathways. Co-delivery of these agents within a single nanoplatform allows simultaneous targeting of these distinct but complementary mechanisms, leading to a more comprehensive and potent approach to inhibiting cancer progression [124,195,196]. This multifaceted strategy takes advantage of what each vitamin does best: vitamin D can effectively “freeze” cancer cells by stopping their growth and encouraging them to mature, while vitamin K can then “trigger” their programmed death [197]. This combined action often results in a significantly enhanced overall tumor regression, surpassing the therapeutic efficacy achievable with either vitamin alone, as demonstrated by numerous in vitro and in vivo studies [16,198,199].

2. Overcoming resistance: Co-delivery offers a promising strategy to overcome resistance common with single-drug cancer treatments. Cancer cells evade therapies through complex mechanisms, including increased expression of drug efflux pumps (e.g., P-glycoprotein), activation of alternative survival pathways, or target mutations [200,201]. By combining vitamins D and K, which work through different molecular targets and signaling pathways, the chances of cancer cells developing resistance to both at the same time are greatly reduced. This multi-pronged approach makes it harder for cancer cells to find ways to escape, helping the treatment stay effective for longer [194,202]. This strategy can be especially valuable for patients who have developed resistance to standard single-drug treatments or even newer targeted therapies, offering fresh options for tough cases. The precise delivery provided by nanomaterials also helps overcome resistance by making sure high levels of both vitamins reach the resistant cancer cells directly [9].

3. The synergy created by co-delivering these agents can boost effectiveness even at lower doses. Preclinical studies often show that the combined impact of two anti-cancer agents working together is greater than just the sum of their individual effects [203,204]. This implies that a nano-formulation co-delivering vitamins D and K could achieve significant anti-cancer effects at lower individual doses of each vitamin than would be required if they were administered separately. This is a critical advantage for two key reasons: it directly helps to mitigate the inherent dose-limiting toxicities associated with each vitamin individually (e.g., the hypercalcemia that restricts high doses of vitamin D or the potential for coagulation interference with high doses of vitamin K) [193,198,205,206]. By broadening the therapeutic window, this approach makes the treatment safer, more tolerable, and ultimately more viable for patients undergoing long-term cancer therapy. Reduced systemic exposure to highly potent agents is a major goal in modern oncology, and synergistic co-delivery via nanocarriers effectively facilitates this objective [207,208].

4. The inherent flexibility and modularity of bio-functional nanomaterials facilitate the creation of truly multimodal therapeutic platforms. Beyond merely co-delivering vitamins D and K, a single nanoparticle can be meticulously engineered to incorporate additional therapeutic modalities, offering a comprehensive and integrated approach to lung cancer treatment [209]. This could include conventional chemotherapy drugs (e.g., paclitaxel and cisplatin, which are common frontline agents in lung cancer treatment), small interfering RNA (siRNA) for the precise silencing of oncogenes or drug resistance genes, or immunomodulatory agents designed to enhance the anti-tumor immune response within the tumor microenvironment [210,211]. For instance, a sophisticated nanoparticle might be engineered to deliver a cytotoxic agent to rapidly reduce tumor burden while simultaneously releasing vitamins D and K to induce apoptosis and prevent angiogenesis, and also carrying an immune checkpoint inhibitor to activate resident T-cells against the tumor [212]. This “all-in-one” approach promises a more potent, comprehensive, and tailored combination therapy with improved targeting, significantly reduced systemic side effects, and enhanced efficacy against the highly complex and heterogeneous nature of lung cancer [41,213].

In A549 xenograft models, for example, research has demonstrated that encapsulating both calcitriol and menaquinone-4 within a single polymeric nanoparticle can result in a 50% larger tumor volume reduction than the free vitamins [214].

In many lung cancer cell lines, this co-delivery strategy consistently yields better results, including enhanced growth inhibition, increased apoptosis, and reduced metastatic potential. Furthermore, in vivo research employing xenograft models has demonstrated markedly reduced tumor growth and elevated survival rates, underscoring the strategy’s potential for synergistic therapy [215].

## 6. Challenges and Future Perspectives in Clinical Translation

Although combining bio-functional nanomaterials with vitamins D and K shows great promise for lung cancer treatment, turning these encouraging early results into everyday clinical use comes with several major challenges. Addressing these will require close collaboration between researchers, clinicians, and regulatory agencies [216,217].

### 6.1. Translational Challenges

Moving nano-vitamin formulations from preclinical research into clinical practice involves several important challenges that must be carefully managed to ensure they are safe, effective, and meet regulatory standards. A key challenge is the ability to scale up manufacturing efficiently [218]. The production of nanomaterials for clinical use demands robust, reproducible, and scalable manufacturing processes. Ensuring batch-to-batch consistency in terms of critical quality attributes, such as size, shape, drug loading, stability, and surface functionalization, is paramount for achieving reliable therapeutic outcomes and meeting stringent regulatory requirements. Current lab-scale synthesis methods may not easily translate to large-scale, GMP-compliant production, posing a considerable challenge in bringing these innovations to patients [219,220,221].

Another critical aspect is the safety and biocompatibility of the nanomaterials themselves. Despite their therapeutic promise, the long-term safety profile of various nanomaterials in humans needs exhaustive evaluation. There are ongoing concerns about immunogenicity—where the body’s immune system might react negatively to the foreign nanoparticles, causing unwanted inflammation or quickly clearing them from the body [13,222]. Systemic toxicity, the buildup of long-lasting breakdown products, and the potential accumulation of nanoparticles in non-target organs—like the liver, spleen, kidneys, or even the lungs after repeated exposure—are important concerns that require thorough toxicology testing. Because nanoparticles interact with biological systems in complex ways that depend heavily on their physical and chemical properties, safety evaluations need to be tailored for each specific formulation [223].

Accurately regulating the pharmacokinetics and in vivo biodistribution of nano-vitamin formulations remains a significant challenge. Although targeted delivery is the main objective, several factors can affect the effectiveness and safety of nanoparticles, including opsonization (the process by which immune cells mark them for destruction), rapid clearance by the reticuloendothelial system, and the formation of a “protein corona” around them once they enter the bloodstream. To completely understand how these formulations act in the body, as well as their absorption, distribution, metabolism, and excretion profiles, to determine effective dosing regimens, comprehensive pharmacokinetic and pharmacodynamic investigations in pertinent animal models are necessary [12,82,224,225].

Navigating the evolving regulatory landscape for nanomedicines is another major challenge. Obtaining approval from agencies like the FDA or EMA for new nano-drug delivery systems demands extensive preclinical data, carefully designed clinical trials, and a thorough understanding of their unique characteristics and risks compared to traditional drugs. This process is often long, complex, and resource-intensive, requiring significant investment and scientific expertise [226,227].

Lastly, the cost of production for many advanced nanomaterials can be substantial due to complex synthesis and purification processes. This economic factor could potentially limit their accessibility and affordability in healthcare systems worldwide, especially in resource-constrained regions, despite their potential benefits. Strategies for cost-effective manufacturing will be crucial for broader clinical adoption [228,229,230].

One of the biggest obstacles to the clinical development of nanomedicine products is the absence of consistent standards. This problem makes it more challenging to scale up production effectively while maintaining a consistent level of product quality. Ensuring batch-to-batch uniformity in terms of size, shape, and stability is challenging without consistent physicochemical characterization and values. These factors are essential for dependable therapeutic effects as well as regulatory approval. Because nanoparticles interact with biological systems in intricate, property-dependent ways that make a universal testing protocol impractical, this regulatory vacuum also makes comprehensive safety evaluations more difficult [231].

Furthermore, the exact control of biodistribution and pharmacokinetics is hampered by the lack of clear criteria for characterization and values, which raises the complexity and expense of the regulatory procedure and delays the application of these advancements to patient care [232].

### 6.2. Clinical Translation and Implementation

For nano-vitamin formulations to transition from promising preclinical results to successful clinical implementation, a carefully orchestrated series of steps is required, emphasizing robust data, meticulous trial design, and a focus on personalized approaches [221,233].

Initially, generating robust preclinical data is paramount before embarking on human clinical trials. This requires thorough and reproducible studies using relevant animal models of lung cancer. These studies need to carefully assess dose–response relationships, show strong effectiveness against different lung cancer types, establish a solid long-term safety profile, and explore potential synergy with current standard treatments like chemotherapy and immunotherapy. This data forms the essential foundation needed to move forward with human clinical trials [234,235].

Furthermore, the successful progression to human trials necessitates well-designed clinical trials. This includes meticulously determining optimal dosing regimens, considering various administration routes (e.g., intravenous infusion, nebulized inhalation for local lung delivery), and carefully stratifying patient populations [236]. Patient stratification using relevant biomarkers—like VDR expression in tumor cells, specific tumor antigens such as EGFR, or molecular pathways involved in vitamin metabolism—is essential to identify those most likely to benefit from nano-vitamin therapies. This approach can improve trial success rates and move us closer to personalized medicine. Early-phase trials, such as Phase 0 and Phase I, will be key for assessing initial safety, pharmacokinetics, and early signs of effectiveness in small groups of patients [237,238].

Also, the identification of biomarkers is inextricably tied to individualized therapy. Finding precise predictive biomarkers that can pinpoint which individuals are most likely to benefit from particular nano-vitamin treatments is essential. In addition to vitamin receptor expression, this also includes indicators of vitamin metabolic pathways, the degree of inflammation in the tumor microenvironment, and other genetic and proteomic signatures associated with a favorable response to treatment. Clinicians will be able to customize therapies for each patient using these biomarkers, optimizing benefits and reducing needless exposures [237,239].

Ultimately, successfully overcoming tumor heterogeneity remains a significant challenge in lung cancer treatment. Lung tumors are highly heterogeneous, both genetically and phenotypically, even within the same patient [240]. A single nano-vitamin formulation, no matter how precisely targeted, might not be effective for all cancer cells within a heterogeneous tumor or across different patient profiles. Future strategies should consider multi-nanocarrier approaches that deliver different therapeutic agents simultaneously or highly personalized designs, where the nano-formulation is customized based on an individual patient’s unique tumor molecular fingerprint. This might involve liquid biopsies to track changes in tumor characteristics over time and adapt the treatment accordingly [12,14,49,198,241].

### 6.3. Future Research Directions

Despite the formidable challenges, the field of nanomedicine for lung cancer therapy is advancing rapidly, driven by innovative research and technological breakthroughs. Several promising future research directions could significantly accelerate the clinical translation of nano-vitamin therapies, building upon the foundational understanding of their synergistic potential [12,50].

To overcome ICI resistance, vitamin D should be used as an adjuvant therapy in addition to the current immunotherapies for lung cancer. The best, most individualized vitamin D dosage plans for patients with lung cancer should be established, taking into account their unique metabolic profiles and the features of their tumors.

The precise processes via which vitamin D affects epigenetics and gene regulation in the TME should be further investigated. Trustworthy biomarkers to direct treatment and forecast response, such as 24-hydroxylase levels and VDR expression, should be created. To enhance overall treatment results, novel combination treatments that combine vitamin D with additional immunomodulatory substances should be investigated.

It is necessary to clarify the function of dietary intake: Further research is required to confirm the direct association between dietary vitamin K and lung cancer risk and outcomes. In-depth mechanistic research is required to understand further how particular types of vitamin K combat cancer at the molecular level.

It is necessary to investigate the possibility of adjuvant therapy. To improve the TME and strengthen existing lung cancer treatments, vitamin K should be investigated as a supplemental medication. Clinical trials should be carried out to assess the effects of vitamin K supplementation on the survival, metastasis, and progression of lung cancer.

The development of biomarkers is necessary in order to track the efficacy of treatment and customize vitamin K interventions.

#### 6.3.1. Development of Responsive Nanocarriers

One key area of focus is the development of smart and responsive nanomaterials. Next-generation nanomaterials are being designed to respond precisely to specific cues within the tumor microenvironment, further enhancing their specificity and minimizing systemic side effects. This includes nanoparticles that can release their cargo in response to the slightly acidic pH found in tumor tissue (pH-responsive release) or those that degrade and release vitamins in the presence of elevated enzyme concentrations (enzyme-responsive degradation) that are characteristic of tumor invasion [19,40,242]. Other exciting possibilities include hypoxia-activated release in low-oxygen tumor regions and light-triggered release, where an external light source (e.g., near-infrared) can induce precise, localized drug liberation, offering exquisite spatiotemporal control over therapy [243,244].

#### 6.3.2. Multifunctional and Theranostic Platforms

Another crucial direction involves the creation of multifunctional nanoplatforms. Beyond the co-delivery of vitamins D and K, future research should explore the synergistic integration of these vitamins with other therapeutic modalities within a single nanocarrier, containing conventional chemotherapy drugs, siRNA for precise gene silencing of oncogenes or drug resistance genes, and immunomodulators intended to increase local anti-tumor immune responses, all customed to attack the complex nature of lung cancer [245,246].

Further development in imaging and theranostics is also vital. This involves creating nano-systems that combine diagnostic imaging capabilities (e.g., fluorescence, MRI, photoacoustic imaging) with therapeutic functions (vitamin delivery, photothermal therapy). These theranostic nanoparticles would enable real-time monitoring of drug delivery and accumulation at the tumor site, track drug release kinetics, and visualize changes in tumor response and disease progression. Such dynamic feedback would empower clinicians to make more informed treatment decisions, adjusting therapy as needed to optimize patient outcomes [198,247].

#### 6.3.3. Personalized Nanomedicine Using Biomarkers and Organoids

The emergence of personalized nanomedicine represents a transformative future. This means using advanced tools like patient-derived organoids—3D cell cultures that closely mimic a patient’s tumor—and powerful artificial intelligence algorithms to design and predict how well nano-vitamin treatments will work for each person’s unique cancer [248]. This approach would move away from a “one-size-fits-all” model towards highly customized treatment strategies based on a patient’s specific genetic mutations, protein expression profiles, and tumor microenvironment, maximizing therapeutic benefit while minimizing off-target effects [198,247].

Finally, exploring the combination with immunotherapy holds immense potential. Both vitamin D and K have been recognized for their immunomodulatory properties. Their targeted delivery via nanomaterials could enhance the efficacy of existing and emerging immunotherapies, such as immune checkpoint inhibitors (e.g., anti-PD-1/PD-L1 antibodies) [12,19]. By locally delivering vitamins D and K to the tumor microenvironment, these nanoparticles could help reprogram immunosuppressive elements, activate anti-tumor immune cells, and render the tumor more susceptible to immunotherapy, potentially overcoming current limitations in response rates for some patients. This synergistic interaction between targeted nutrient delivery and immune modulation represents a highly promising avenue for future lung cancer therapy [19,249].

## 7. Conclusions

Lung cancer remains one of the toughest health challenges worldwide, pushing researchers to find newer, more effective treatments. Recently, bio-functional nanomaterials have been changing the game in drug delivery. They allow medicines to hit their target precisely, release drugs in a controlled way, and even combine different therapies all at once. Meanwhile, vitamins D and K, which are fat-soluble, have shown real potential in fighting cancer. They work by slowing down the growth of cancer cells, prompting them to die, controlling blood vessel growth, and stopping tumors from spreading.

However, it has not been simple to use these vitamins directly in therapy. The situation has become complex due to issues like inadequate absorption, rapid bodily breakdown, and unintended side effects outside of the tumor. Combining these vitamins with nanomaterials is one way to address that. Vitamins D and K dissolve more readily and remain stable when enclosed in specially made nanocarriers. It also helps the vitamins gather in tumor sites more effectively, thanks to both natural and targeted delivery methods. This approach not only makes the vitamins more powerful against cancer but also cuts down on side effects, making treatments safer and more effective.

On the one hand, vitamin D metabolism impairment in lung cancer patients is frequently exacerbated by factors like smoking, which is known to speed up the breakdown of active vitamin D. Vitamin D is often inactivated by lung cancers through overexpression of CYP24A1, with active circulating vitamin D levels being lowered as a result. Lower serum vitamin D levels are correlated with advanced stages of lung tumors.

The immunosuppressive tumor microenvironment (TME) is significantly altered by vitamin D, which stimulates T-cell activation and controls immunological checkpoints such as PD-1. Immune Checkpoint Inhibitors (ICIs) become more effective as a result, indicating that they may be used in concert with other immunotherapies already in use. Importantly, tumor cells can be made more sensitive to the immune system by vitamin D, giving new hope to patients with lung cancer who have become resistant to immunotherapy.

On the other hand, the connection between vitamin K and cancer is still being established. The spread of cancer cells may be prevented by vitamin K through the reduction in inflammation (for example, by decreasing IL-6 and TNF-α). The active forms (K2, K3) are seen to have potential: anti-cancer ROS may be generated by K3, while NF-κB, which is essential for tumor growth, is suppressed by K2. By reducing oxidative stress and inflammation, which are essential for the growth and metastasis of cancer, the tumor microenvironment is helped to be modified by vitamin K. Vitamin K mainly is considered to act as an anti-inflammatory, which is known to help immunity, particularly in inflammatory and malignant conditions.

Undoubtedly, there are difficulties in transferring these vitamin-loaded nanoparticles from the laboratory bench to actual patient treatment. The process includes increasing output, ensuring long-term safety, and overcoming regulatory obstacles. However, there is real promise thanks to developments in nanotechnology and a greater understanding of how these vitamins prevent cancer. It will be crucial to continue refining vitamin combinations, enhancing nanomaterial designs, and conducting extensive laboratory and clinical testing.

Bringing together nanomedicine and vitamin-based treatments is an exciting, promising approach. It shines a new light on the fight against lung cancer and brings us closer to a future where this devastating disease can be managed—and potentially overcome.

The combination of nanomedicine and vitamin-based therapies represents a powerful approach. It brings new optimism to the fight against lung cancer and moves us closer to a future where this devastating disease can be effectively controlled and even overcome.

## Figures and Tables

**Table 1:** Bio-functional nanomaterials: principles and applications in oncology.

Principle	Description	Advantages	Disadvantages
Passive targeting	Nanoparticles accumulate preferentially in solid tumors due to leaky tumor vasculature and impaired lymphatic drainage. The size of nanoparticles (20–200 nm) is key [12,35].	Simplifies targeting, as no specific surface modification is required. Leads to higher drug concentrations in the tumor, reducing systemic toxicity [36].	Heterogeneity of EPR effect across tumors and patients. It can be influenced by tumor type, size, and location. Some tumors may have less leaky vasculature, limiting efficacy [37].
Active targeting	Nanomaterials are surface-modified with specific ligands that bind selectively to receptors overexpressed on cancer cells or tumor microenvironment components. Ligand-receptor binding often triggers internalization [12,38].	Enhances specificity and cellular uptake into cancer cells, leading to higher intracellular drug concentrations. Can overcome limitations of heterogeneous EPR. Enables personalized therapy based on biomarkers [37,39].	Potential for off-target binding to healthy cells expressing the target receptor at lower levels. It can be challenging to maintain ligand stability in vivo. May face immune responses against targeting ligands [12,24].
Controlled and stimuli-responsive release	Drug release kinetics are precisely controlled, ranging from sustained release to liberation triggered by specific endogenous cues (pH, enzymes, hypoxia, redox potential) or exogenous cues (light, magnetic fields, ultrasound) in the tumor microenvironment [40,41].	Optimizes drug concentration at the tumor site for longer durations. Minimizes premature drug release and systemic side effects. Allows for on-demand drug release [12,42].	Complexity in designing and synthesizing responsive materials. Potential for incomplete release. Variability in tumor microenvironment conditions can affect the predictability of release [43].
Multimodal therapy and diagnostics	Nanomaterials are engineered to incorporate multiple therapeutic agents (combination therapy) and/or diagnostic imaging agents, enabling simultaneous treatment and real-time monitoring [13].	Offers comprehensive treatment strategies by combining therapies. Enables real-time visualization of drug delivery and therapeutic response. Facilitates personalized medicine and dynamic treatment adjustments [44,45].	Increased complexity in design, synthesis, and characterization. Potential for interaction between different functionalities. Higher regulatory hurdles due to dual diagnostic and therapeutic roles [13].

**Table 2:** Summary of clinical limitations of vitamin D and K treatments.

Limitations		
Vitamin D	Shared	Vitamin K
- Heterogeneous outcomes in supplementation studies, some showing no significant benefits despite correcting the deficiency. - Excessive supplementation may cause toxicity (hypercalcemia and kidney dysfunction). - Uncertainty in optimal dosing for prevention vs. treatment.	- Poor bioavailability due to lipophilic nature. - Rapid metabolism and short half-life, frequent intake required. - Variability in individual responses and lack of consensus on optimal dosing and treatment duration. - Limited long-term clinical trial evidence for efficacy.	- Evidence for benefits beyond coagulation (e.g., bone, cardiovascular, cancer) is inconsistent. - Forms K1 vs. K2 vary in bioavailability and effects, complicating therapeutic use. - Risk of interactions with anticoagulant drugs (e.g., warfarin), restricting use in some patients.
